# Phylogenetic relationships and genetic diversity of Tunisian maize landraces

**DOI:** 10.1371/journal.pone.0316185

**Published:** 2025-01-22

**Authors:** Mohamed Dhia Eddine Hammami, Delphine Madur, Zayneb Kthiri, Agustin Galaretto, Stéphane D. Nicolas, Alain Charcosset, Valérie Combes, Chahine Karmous, Pedro Revilla

**Affiliations:** 1 Laboratory of Genetics and Cereals Breeding, National Institute of Agronomy of Tunisia, Carthage University, Carthage, Tunisia; 2 Misión Biológica de Galicia (Spanish National Research Council, CSIC) Apdo 28, Pontevedra, Spain; 3 Université Paris-Saclay, INRAE, CNRS, AgroParisTech, GQE—Le Moulon, Gif-sur-Yvette, France; Government College University Faisalabad, PAKISTAN

## Abstract

Based on history, maize was first introduced into Tunisia and northern Africa, at large, from the south of Spain. Several subsequent introductions were made from diverse origins, generating new landraces by recombination and selection for adaptation to arid environments. This study aimed to investigate the phylogenetic relationships among Tunisian maize landraces with possible sources of introduction from neighboring countries. Ten Tunisian landraces were genotyped with 23656SNPs along with a panel of diversity of 171 landraces originating from Algeria, Europe, and America. The Tunisian maize landraces were very diverse and distinct from those from neighboring countries, and they were classified into three main clusters that could be the basis for investigating heterotic groups. The phylogenetic relationships among Tunisian and other landraces from neighboring countries supported the hypothesis of a first introduction from the south of Spain and subsequent introductions from other countries. These germplasm groups could be the basis for studying heterotic patterns and sample maize germplasm from Tunisia and North Africa in general. The Tunisian maize germplasm could be a basis for identifying sources of favorable alleles to improve tolerance to abiotic stresses.

## Introduction

Maize (*Zea mays* L.) was taken outside of America for the first time in 1494, when Columbus brought it to Seville, in southern Spain [1,2]. Afterwards there were successive introductions of maize in southwestern Europe. Subsequently, maize was introduced to the rest of the Old World by European explorers and traders [1,2]. The introduction to North Africa could have been made by Spanish Muslims who traveled through the Muslim empire that spanned from northwest Africa to southeast Asia [2]. The large distribution of maize throughout the World has generated a huge genetic diversity that provides favorable alleles for breeding maize for adaptation to specific environments and to promote sustainable production of food under changing environmental conditions [3].

Historical data about maize introduction in northern Africa are not conclusive, but Grigg [4]

proposed that maize was introduced by the Turks in Egypt soon after the invasion in 1517. We cannot be sure about the first introductions, and the evidences of maize cultivation are not significant until the seventeenth century. For example, according to Hafnagel [5] maize was introduced in Ethiopia by the seventeenth century, which was the time by which it was introduced in northwestern Spain, according to Pérez-García [6]. Finally, Bouza-Brey [7] assumes that maize was in Morocco by 1531 and was cultivated in France before the seventeenth century. As soon as maize became a popular crop in southern Spain, it could have been spread through the Muslim empire by traders, sailors, warriors, or pilgrims going to the Mecca from Spain and back home to southeastern Asia or any other area in the vast intercontinental Muslim territory.

Rebourg et al. [8] also confirmed that maize was first introduced in southern Spain from the Caribbean area, and shortly after from North America through the European Atlantic coast. These two introductions originated the two main European maize pools, i.e. the Mediterranean and the European flints, respectively, which were crossed afterwards and selected for adaptation to diverse environments and uses. Gauthier et al. [9] classified maize landraces from Europe by using molecular markers and found three main clusters consistent with geographic origins. First a Northeastern cluster, then a southeastern cluster with semident landraces from southeastern Europe, and finally a southwestern cluster with early flint landraces from northwestern Europe. They also detected a possible direction of gene flow indicating that Northern flint and Caribbean landraces were introduced to northern and southern Europe, respectively. The maize’s genetic structures were categorized based on geographic origins and genetic diversity [10], who identified significant diversity within European and American landraces, discriminating them into specific groups such as Northern Flint, Pyrenees-Galicia Flint, Italian Flint, Mexican, Caribbean, and Andean landraces. Camus-Kulandaivelu et al. [11] propose also the same classification of maize genetic structures based on their association with flowering time, with Northern Flint, Pyrenees-Galicia Flint, Italian Flint, Corn Belt Dent, and the Tropical group, which includes Mexican, Caribbean, and Andean varieties reflecting varying levels of divergence from the ancestral maize pool.

The introduction of maize in Africa has been scarcely studied from a genetic perspective, but the available genetic data indicate that the previous hypothesis makes sense [12,13]. Mir et al. [14] found that genetic relationships among maize from the Old World and American maize were consistent with historical data, showing that maize was introduced in Europe and Africa from diverse American origins. Therefore, maize was first introduced from the Caribbean area into southern Spain and Portugal, and subsequently through the Mediterranean area, which was the first source of maize diffusion across the Muslim empire in Africa and Asia. They also found that maize from Morocco, Algeria and Italy were genetically close and that maize from North America was introduced by Portuguese and Spanish travelers from the sixteenth century. Aci et al. [13] corroborated that maize diversity in Algeria was consistent with three main introductions from southern Spain, African neighbor countries and later introductions from Europe. Indeed, Aci et al. [13] grouped 15 Algerian maize landraces by using molecular markers and found three groups relatively consistent with geographical origin. The first group included only one landrace from the most southern area. The second cluster consisted on nine landraces from the center of Algeria and was divided in two subgroups. The third cluster had five landraces from northern areas of Algeria.

The most historically consistent hypothesis of the introduction of maize into Tunisia is that maize from southern Spain and the subsequent Mediterranean pool was the first source, followed by later introductions in which European flint and combinations of different maize origins may have participated. Nevertheless, the phylogenetic diversity of maize in Tunisia has not been reported. Our objectives are 1) to verify whether the genetic diversity among Tunisian maize landraces is consistent with the previously postulated hypothesis on the origin of maize in North Africa from Spanish and neighboring countries, and 2) to classify Tunisian germplasm into genetic groups that could be the basis for future investigations of possible heterotic groups in Tunisian maize.

## Materials and methods

### Plant material

We sampled 10 Tunisian landraces from six different bioclimatic regions to represent maize diversity adapted to diverse climatic conditions, from sub-humid to arid areas (S1 Table). We compared their genetic diversity with 156 landraces representing European and American genetic diversity that have been previously analyzed and genotyped with SNP 50K array by Arca et al. [10] and with SSR by Mir et al. [14]. Fifteen Algerian landraces were also included in the comparison with Tunisian populations to explore potential phylogenetic connections between the southern and northern Mediterranean regions and to trace their origins [15]. The Algerian landraces were provided by the North Central Regional Plant Introduction Station of the USA. Each landrace was represented by a DNA bulk that have been extracted from 15 individual plants [16,17]. For each landrace, an equal mass of plant leaves was punched to create the DNA bulk prior the DNA extraction.

### Genotyping and estimation of allelic frequencies in DNA bulks

DNA bulks of 10 Tunisian and 15 Algerian landraces were genotyped with 50K Illumina Infinium HD array [18] and gathered with 156 landraces already genotyped to obtain allelic frequencies matrices of 23 656 SNP according to the procedure described in [19]. Briefly, genotyping analysis was first set up using the Genome Studio Genotyping Module software (v2010.2, Illumina lnc) to extract the Fluorescent Intensity Signal for A and B alleles of each SNP put on the array. The initial set composed of 49,574 SNPs was filtered for their suitability for allelic frequency prediction following the procedure described in Arca et al., 2021, resulting in a final set of 23,656 SNPs.

The allelic frequencies of 23,656 SNPs within DNA bulks were estimated using a two-step approach. Initially, they distinguished between monomorphic and polymorphic SNPs by comparing DNA bulks’ fluorescent intensity ratios (FIR) against those of inbred lines. For SNPs identified as polymorphic, a unique logistic model was calibrated using FIR data obtained from 1,000 SNPs in two sets of controlled pools with known allelic frequencies to estimate the frequency of the B allele [19]. This approach achieved a conservative estimation for SNPs near fixation with a mean absolute error of 3% and set a 5% error threshold to reject the hypothesis of monomorphism in landraces [19].

### Genetic distance structure and interrelationships among landraces

After replacing the missing genotyping data by the mean allelic frequency of each locus, the modified Rogers distance (MRD) [17,20] was calculated based on the allelic frequencies of 23,656 selected SNPs between 181 landraces. The 23,656 filtered SNPs were used to examine the structure of genetic diversity within the landraces panel through two methods: (i) a distance-based approach by generating a principal Coordinate Analysis (PCoA) [21] and a Hierarchical clustering employing Neighbour-Joining and Ward algorithms, implemented in the ’bionj’ and ’hclust’ functions of the ’ape’ R package v 5.0 [22]. (ii) Genetic structure prediction was performed by the R package quadprog used to adjust linear model coefficients and put a constraint for the sum of coefficients to one [23]. This approach probabilistically assigned each landrace to K ancestral populations and employed penalized linear regression on the genotyping data of the 156 landraces for which the ancestral genetic groups have been defined beforehand [24].

The linear penalized regression aims to predict a percentage of belonging to the different ancestral groups for each of the 181 landraces to 7 genetic groups established on 156 landraces representing European and American diversity, using the mean allelic frequency matrix for each genetic group [10].

## Results and discussion

The genetic diversity (expected heterozygosity, Hs, and number of monomorphic loci, NbMono) of some Tunisian landraces, such as Bk or MT2, was similar to reference populations like Reid Yellow Dent (Hs = 0.2662, NbMono = 4043) or Lancaster Sure Crop (Hs = 0.2670, NbMono = 3556) (S1 Table). However, most Tunisian landraces were below those values of Hs and NbMono.

**Fig 1:**
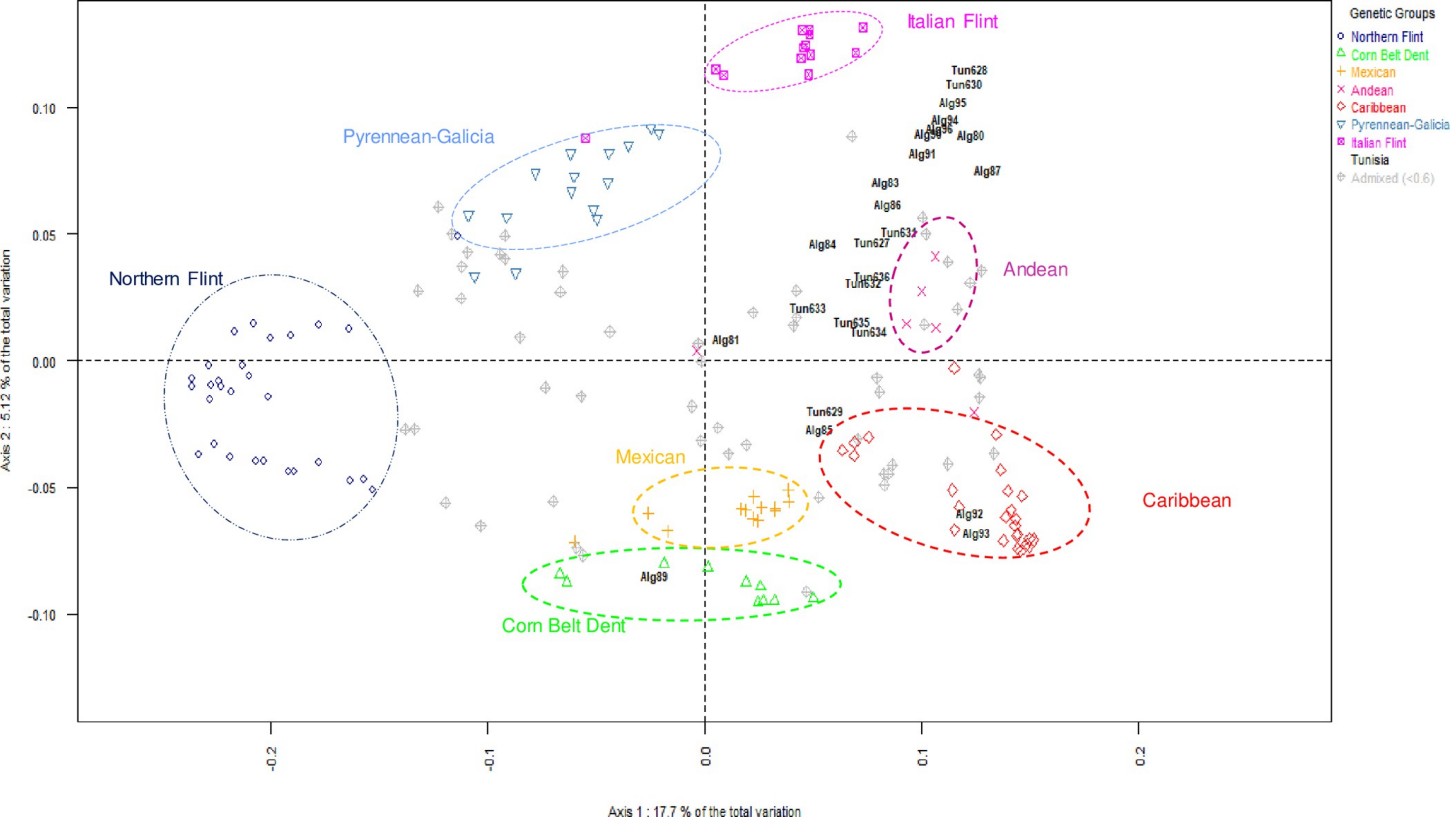
Principal Coordinate Analysis (PCoA) of the genetic diversity among of 191 populations from Europe, America and North Africa based on their modified Roger’s distance (MRD).

**Fig 2:**
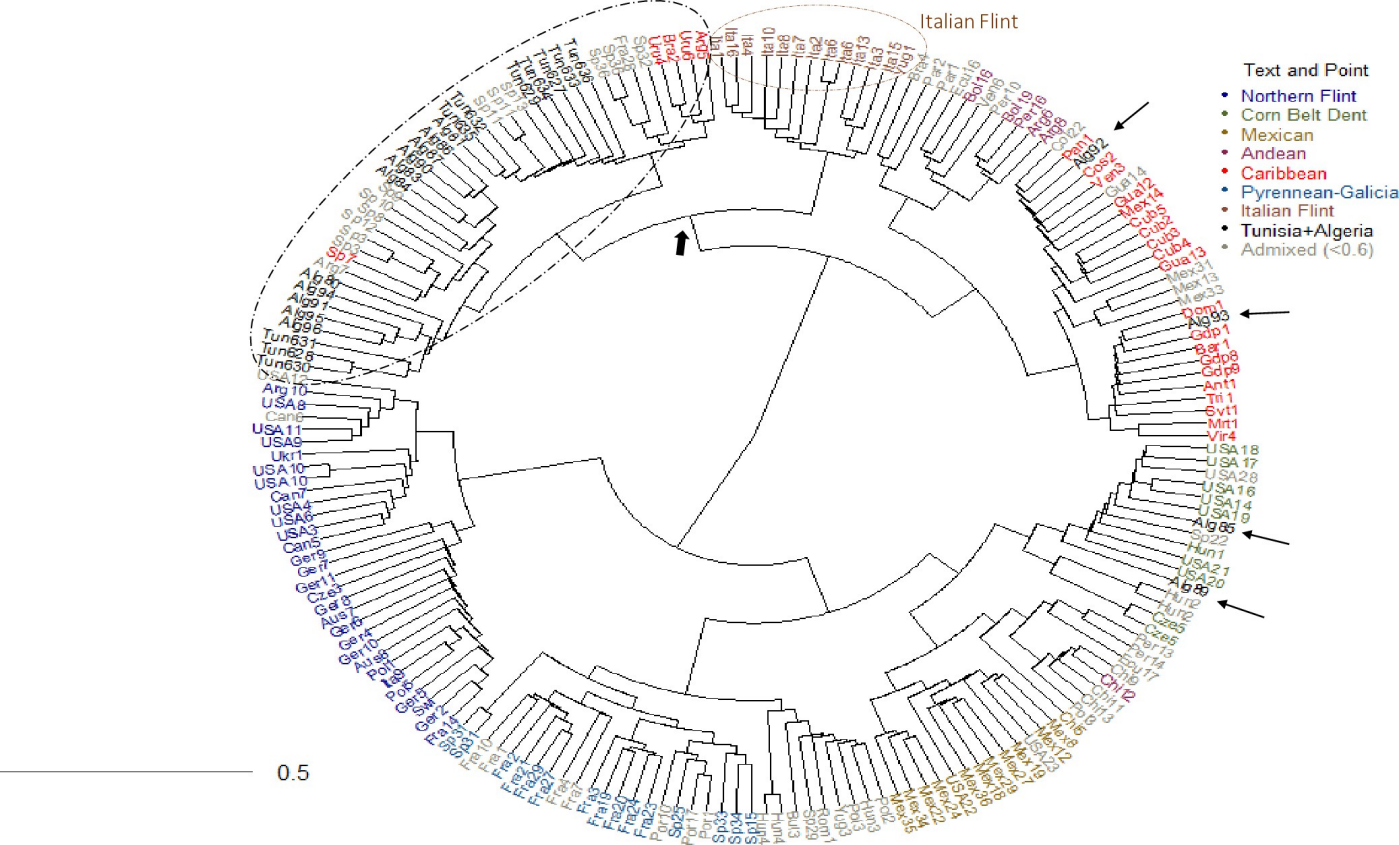
Dendrogram of 191 maize populations from nine origins, namely Northern Flint, Corn Belt Dent, Mexican, Andean, Caribbean, Atlantic European coast (Pyrenean-Galicia), Mediterranean (Italian flint), Algeria and Tunisia, made by neighbor joining using the modified Roger’s distance (MRD).

The relationship between maize landraces was investigated using Principal Coordinate Analysis (PCoA) (Fig 1) and Neighbor joining hierarchical clustering based on MRD (Fig 2). The PCoA showed that the first axis (Axis 1), representing 17.7% of the total variation, discriminated Northern Flint landraces from tropical and subtropical. The second axis (Axis 2), capturing 5.12% of the total variation, opposed the Corn Belt Dent and Mexican landraces from the Italian Flint and Pyrenean-Galician landraces.

The PCoA revealed that the Tunisian and Algerian landraces were distant from the Italian-Flint, Northern Flint, and Pyrenean-Galicia; but they were closer to the Andean and Mexican groups, some Algerian landraces were even included in the Caribbean, and Corn Belt Dent groups, and they were among some admixed landraces (Fig 1). The Tunisian Landraces were closer to Italian-Flint and Andean landraces, while Algerian landraces were more related to the Caribbean group.

None of the Tunisian landraces was included in any of the groups of germplasm previously defined by [10]. The Tunisian landraces Biz2 (Tun 628) and Biz1 (Tun 630) were close to the Italian Flint cluster. This is highly consistent with the results of penalized regression analysis in which Biz2 (Tun 628) and Biz1 (Tun 630) from Bizerte were mainly assigned to Italian Flint, respectively with 57% and 56%. At the same time, seven other Tunisian landraces are admixed between Italian Flint and Caribbean (S2 Table). The Algerian landraces Alg92 and Alg93 were located within the Caribbean group, and Alg89 within the Corn Belt Dent group. Besides, Alg85 and Tun629 were close to the Caribbean group according to the plot of the two main principal components (Fig 1), though most of them were closer to the Andean group. Several Algerian landraces were mixed with the Tunisian landraces in that plot.

The hierarchical clustering of 25 North African landraces with 156 European and Mexican landraces revealed four prominent clusters composed respectively of, Northern-Flint landraces, Pyrenean-Galicia with Mexican and Corn Belt Dent landraces, South American and Andean landraces, and the last one containing the Tunisian and Algerian landraces; that group was composed of two sub-clusters the first one contained the Italian-Flint, the second group clustered the Tunisian and Algerian landraces except Alg92, Alg93, Alg85 and Alg89S with Spanish, French and South American landraces (Fig 2).

**Fig 3:**
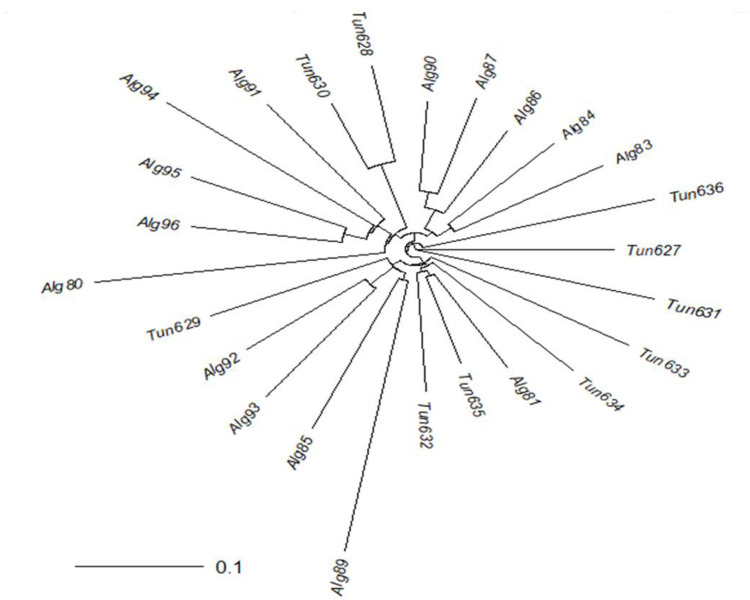
Dendrogram of 15 landraces from Algerian and 10 from Tunisian, made by neighbor joining based on their modified Roger’s distance.

Tunisian landraces were classified into two main sub-clusters. The first sub-cluster was constituted by the landraces Djerba (Tun636), GAF (Tun633), Gab1 (Tun627), KAR (Tun634), and MT1 (Tun629), with MT2 (Tun632) and BK (Tun635), and formed a group with Basto (Sp11) and Tremesino (Sp13), which are landraces from the eastern and southern parts of Spain, i.e., from the dry Mediterranean area. In addition to Italian-Flint, the landraces (SP36), (SP28), and (FRA28), respectively, from the humid north of Spain and France, and the South American landraces (ARG5), (Uru6), (Bra2), and (Uru4) were also close to the Tunisian landraces. The Algerian landrace PI527465 (Alg81), from Al Ehdira (Gahardaia), was very close to BK (Tun635) followed by MT2 (Tun632) (Figs 2 and 3). The second sub-cluster was constituted by Biz1 (Tun630), Biz2 (Tun628), and Gab2 (Tun631) with close Algerian landraces such as (Alg91) from the province of Bechar, Alg94, Alg95 and Alg96 from Tamanrasset and Alg80 from Ouargla. Biz1 (Tun630) and Biz2 (Tun628) exhibited a strong association. Besides the Italian landraces, the Algerian and Tunisian landraces of the second subcluster were close to Spanish landraces such as Utrera (Sp8), Villamartín (Sp7), Andaluz (Sp3,) Andaluz_ABC (Sp12), Pabilillo de Granada (Sp9) and Blanco de Ricote (Sp10), which are from the dry Mediterranean Spain and (Arg7) from the subtropical region of Argentina.

The closest European landraces to the Tunisian and Algerian landraces were Basto (Sp11), and Tremesino_ABC (Sp13) from the dry Mediterranean area of Spain. This suggests that the Tunisian and Algerian landraces may originated from the Spanish landraces, which is consistent with the trades conducted by Islamic merchants. Four Algerian landraces were apart from this cluster. Alg85 originated from Timimoum in the province of Adrar, PI527472 (Alg85), and PI527477 (Alg89) originated from Bechar clustered with the Corn Belt Dent Landraces from Central American and European countries. Alg92 (PI542684) and Alg93 (PI542685) originated from Ideles and Mertoutek, respectively, clustered with Caribbean accessions from Cuba and other Central American countries.

In the dendrogram of the Algerian and Tunisian landraces (Fig 3), MT1 (Tun629), from the region of Nabeul, was isolated from the other Tunisian landraces and in the same group as three landraces from the Algerian province of Bechar (Alg92, Alg93, and Alg89) and one from Adrar (Alg85). This group was far from MT2 (Tun632) and BK (Tun635), the two other Tunisian landraces from Nabeul, which were close to KAR (Tun634) from Kairouan and GAF (Tun633) from Gafsa, close to the Algerian border. At the same time, that group was also near an Algerian landrace from the province of Ghardaia (Alg81) (Fig 2). On the other side, BIZ2 (Tun628) and BIZ1 (Tun630), the Tunisian landraces from Bizerte, were close and in the same group as one Algerian landrace from the province of Bechar (Alg91), three from Tamanrasset (Alg94, Alg95 and Alg96), and one from Ouargla (Alg80), along with other Algerian landraces. Finally, the landraces Gab2 (Tun631), Gab1 (Tun627), and Djerba (Tun636), from the two closest Tunisian southern regions of Gabes, and the island of Djerba were neither related among them, nor with the other Tunisian or Algerian landraces. The most plausible hypothesis to explain this diversity of relationships is the multiple introductions previously hypothesized [1,2,9,12,13].

Therefore, the two main groups of Tunisian landraces are MT1 (Tun629), MT2 (Tun632), GAF (Tun633), KAR (Tun634), and BK (Tun635) from three different regions (Nabeul, Gafsa, and Kairouan); and the landraces from Bizerte BIZ2 (Tun628), and BIZ1 (Tun630). The landraces from Gabes Gab1 (Tun627), Gab2 (Tun631), and Djerba (Tun636) are part of a third group, although they are not closely related to each other. These three groups should be the basis for exploring possible heterotic patterns among Tunisian landraces and heterotic relationships between Tunisian landraces and heterotic groups from temperate areas of Europe and elsewhere. Since the Tunisian landraces clustered together with the Algerian landraces, it is also interesting to consider the possible Algerian heterotic patterns. Although Aci et al. [13] used another group of Algerian landraces, they found that a landrace from Tamanrasset, in the far south, was genetically isolated. They found also a cluster with nine landraces from Adrar and the North of Algeria, along with a southern landrace. Finally, a third cluster included landraces from Bechar. The geographical distribution of Algerian landraces was not completely consistent with the genetic distance. These clusters were also consistent with the hypothesized introductions from the original Spanish sources, other African neighbor countries and later introductions from Europe.

**Fig 4:**
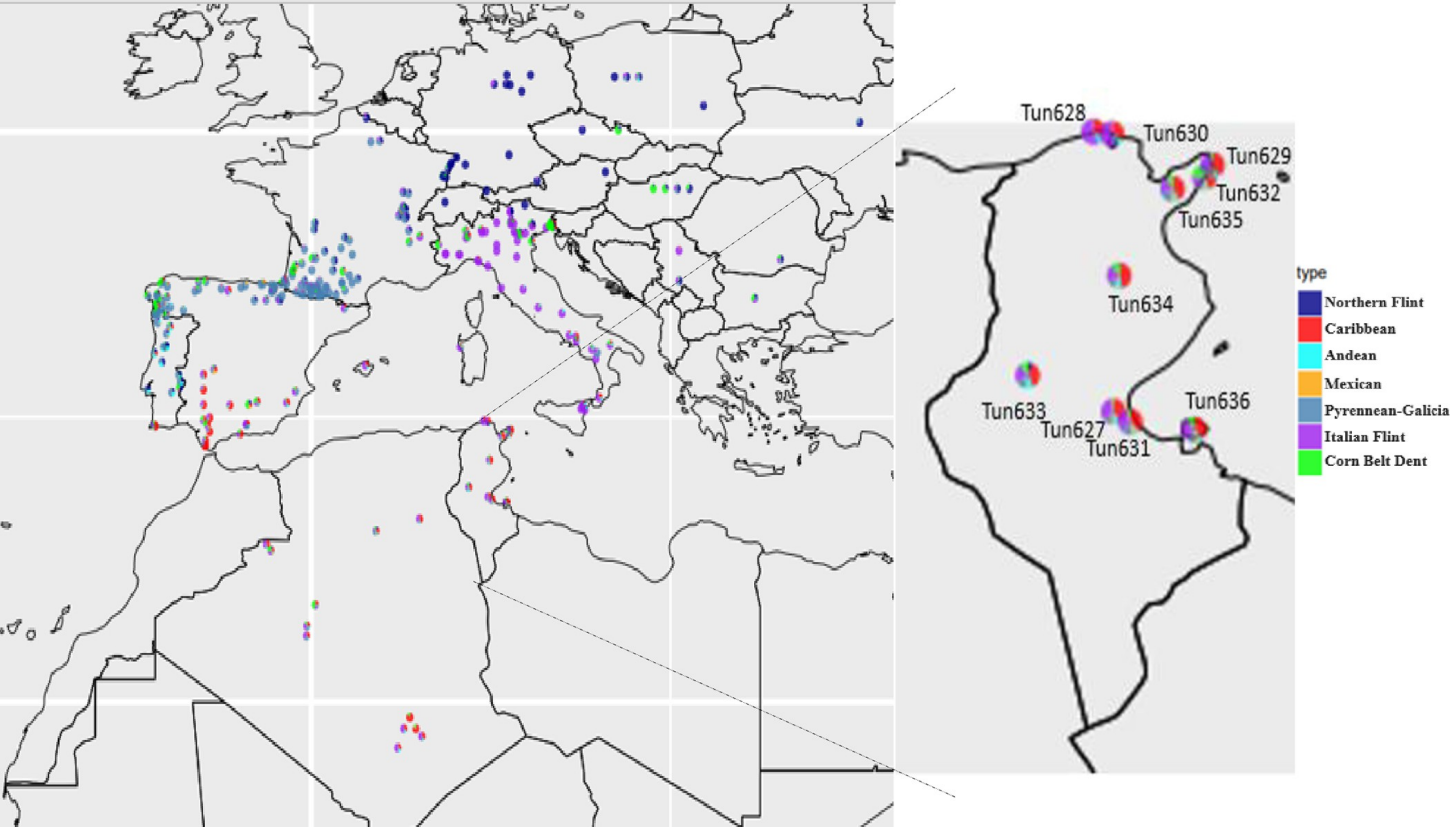
Spatial genetic structuration of North Africa Mediterranean maize and ten Tunisian maize landraces compared to European maize landrace from Mediterranean countries.

The genetic similarities among the maize landraces within this Mediterranean area are consistent with the geographic origin of the landraces (Fig 4). This map shows that Italian germplasm is distinct from neighbor European countries but is related to Algerian and Tunisian landraces, which is consistent with geographical proximity of Tunisia and Algeria from Italy. Maize genetic groups are clearly distinct in Tunisia, probably because it has clearly distinct climatic areas, even though it is a small county (163000 km^2^).

The assignment of Tunisian landraces to the seven genetic groups (S2 Table) revealed that Tunisian landraces were mainly assigned to the Caribbean (38.2%) and Italian Flint (32.6%) while they are weakly assigned to Northern Flint (1.5%), Mexican (3.1%). Two landraces, Biz2 (Tun 628) and Biz1 (Tun630), could be assigned to the Italian Flint Group, representing 57% and 56% of the total genetic variability. This finding could be explained by the short geographical distance between Bizerte, the region where these two landraces are from, and Italy. For the Caribbean group, KAR (Tun634) followed by GAB2 (Tun631), MT2 (Tun632), and BK (Tun635) were the landraces plus assigned to this group, with 44% for Tun634 and 41% for the other landraces. Most of the Tunisian landraces are not assigned to the Northern Flint group (S2 Table), except for three landraces: KAR (Tun634), MT1 (Tun 629), and GAF (Tun633), registering a low similarity, respectively, with 2%, 5%, and 8% of the total genetic variability.

The close relationships between the two Tunisian landraces Biz2 (Tun 628) and Biz1 (Tun630) is supported by their similar level of assignment to Caribbean and Italian groups, and a small Andean contribution, while the rest of Tunisian landraces are more diverse (S2 Table). Probably, the Tunisian landraces come from a relatively large number of sources of germplasm. This is also the case for Gab1 (Tun627), Gab2 (Tun631), as these two landraces show genetic similarities with Caribbean and Italian germplasms, but also with maize from the European Atlantic coast (European) and some minor sources. The Caribbean contribution is important for all Tunisian landraces, which agrees with the hypothesis of the original introduction from the Caribbean area [1,2,8]. Also, the Italian contribution is important in most landraces, probably due to the short distance between Tunisia and Italy. As Tenaillon and Charcosset [3] pointed out, the introduction of diverse sources of germplasm in a new environment, followed by recombination of the diverse genetic backgrounds and selection for adaptation to environments ranging from the desert to the humid Mediterranean coast, has produced potential sources of favorable alleles for improving tolerance to the specific stresses.

These results are consistent with the hypothesis about an original introduction of maize in North Africa from the Caribbean region [12,13]. Furthermore, the relationship between Algerian and Tunisian landraces is consistent with the hypothesis of maize introduction in the north of Africa by Spanish Muslims because some Tunisian landraces were genetically close to the landraces from Algeria and the south of Spain [2,14]. On the other hand, the relationship among a landrace from the Spanish Atlantic coast and the Tunisian landraces can be explained by a common origin or later movements of maize by traders. The two main clusters of Tunisian landraces are consistent with the geographical distribution, as the cluster of Tun628 and Tun 630 comes from the north coast of Tunisia, while the large group includes the populations from the mountains. Finally, the three single-landrace clusters were collected in arid areas.

## Conclusions

There were two main clusters of Tunisian maize landraces, one of them with large genetic diversity, and three landraces that were genetically different. The relationships among Tunisian landraces and other landraces from neighbor countries were consistent with the hypothesis of a first introduction from the south of Spain and subsequent introductions from other countries, particularly Italy. These clusters could form the basis for future studies about heterotic patterns and representative sampling of maize germplasm from Tunisia and North Africa in general. The Tunisian maize germplasm constitute novel and unique germplasm pools that come from crosses and recombination of diverse European and American sources and has been adapted by selection to adaptation to diverse Mediterranean climatic conditions.

## Supporting information

S1 Table Regions, bioclimatic zones and genetic diversity of Tunisian maize landraces included in this study. (DOCX)

S2 Table Assignment of Tunisian maize landraces to genetic groups based on genotypic analysis made with 23656 SNPs. (DOCX)

S3 Table (CSV)

S1 File (TXT)

S2 File (TXT)

S3 File (TXT)

S4 File (TXT)

S5 File (TXT)
