# Supplementary material for: Phylogenetic relationships and genetic diversity of Tunisian maize landraces
Source: PLoS One. 2025 Jan 22;20(1):e0316185. doi: 10.1371/journal.pone.0316185 (PMC11753626; doi:10.1371/journal.pone.0316185)
Supplement: S1 Table — (DOCX) [file pone.0316185.s001.docx]

| **Code_**  **Mir** | **Landrace_**  **ID** | **Region** | **Latitude** | **Longitude** | **Bioclimatic Zone** | **Hs**^1^ | **NbMono_pop**^2^ |
| --- | --- | --- | --- | --- | --- | --- | --- |
| **Tun627** | GAB1 | Gabes | 33°52'39.3"N | 10°03'53.0"E | Semi-arid with cold winter | 0,2019 | 8429 |
| **Tun628** | BIZ2 | Bizerte | 37°19'45.1"N | 9°36'00.0"E | Subhumid | 0,1712 | 9357 |
| **Tun629** | MT1 | Nabeul | 36°47'11.7"N | 10°59'52.2"E | Subhumid | 0,2412 | 4763 |
| **Tun630** | BIZ1 | Bizerte | 37°18'10.7"N | 9°49'57.0"E | Subhumid | 0,1996 | 7265 |
| **Tun631** | GAB2 | Gabes | 34°00'00.0"N | 9°51'00.0"E | Semi-arid with cold winter | 0,1485 | 11639 |
| **Tun632** | MT2 | Nabeul | 36°55'48.0"N | 11°06'00.0"E | Subhumid | 0,2568 | 3873 |
| **Tun633** | GAF | Gafsa | 34°25'49.9"N | 8°46'05.9"E | Arid with cold winter | 0,2050 | 9799 |
| **Tun634** | KAR | Kairouan | 35°36'57.0"N | 9°55'23.2"E | Semi-arid with cold winter | 0,2250 | 5983 |
| **Tun635** | Bk | Nabeul | 36°38'54.1"N | 10°35'50.4"E | Subhumid | 0,2834 | 2405 |
| **Tun636** | Djerba | Djerba | 33°46'59.7"N | 10°52'10.7"E | Semi-arid with cold winter | 0,1755 | 10192 |

**S1 Table: Regions, Bioclimatic Zones and genetic diversity of Tunisian maize landraces included in this study**

^1^ Expected hetetozygosity

^2^ Number of monomorphic loci
