# Supplementary material for: Phylogenetic relationships and genetic diversity of Tunisian maize landraces
Source: PLoS One. 2025 Jan 22;20(1):e0316185. doi: 10.1371/journal.pone.0316185 (PMC11753626; doi:10.1371/journal.pone.0316185)
Supplement: S2 Table — (DOCX) [file pone.0316185.s002.docx]

**S2 Table:** **Assignment of Tunisian maize landraces to genetic groups based on genotypic analysis made with 23656 SNPs**

|  | **Genetic Groups (%)** | | | | | | |
| --- | --- | --- | --- | --- | --- | --- | --- |
| **Tunisian Landrace** | **Northern Flint** | **Caribbean** | **Andean** | **Mexican** | **Pyrenean Galicia** | **Italian Flint** | **Corn Belt**  **Dent** |
| **Tun627** | 0 | 39 | 5 | 4 | 13 | 35 | 4 |
| **Tun628** | 0 | 33 | 9 | 0 | 1 | 57 | 0 |
| **Tun629** | 5 | 39 | 5 | 4 | 8 | 15 | 24 |
| **Tun630** | 0 | 33 | 9 | 0 | 2 | 56 | 0 |
| **Tun631** | 0 | 41 | 7 | 4 | 10 | 35 | 3 |
| **Tun632** | 0 | 41 | 7 | 4 | 15 | 29 | 4 |
| **Tun633** | 8 | 34 | 11 | 0 | 13 | 23 | 11 |
| **Tun634** | 2 | 44 | 8 | 3 | 10 | 23 | 10 |
| **Tun635** | 0 | 41 | 6 | 6 | 17 | 22 | 8 |
| **Tun636** | 0 | 37 | 6 | 6 | 11 | 31 | 9 |
| **Mean** | 1,5 | 38,2 | 7,3 | 3,1 | 10 | 32,6 | 7,3 |
